# Nanosilicates Enhanced Periodontal Angiogenesis by Regulating Microtubule Dynamic‐Mediated STAT3 Pathway

**DOI:** 10.1111/cpr.70143

**Published:** 2025-11-04

**Authors:** Lingling Shang, Yuhan Hu, Shaohua Ge

**Affiliations:** ^1^ Department of Periodontology, School and Hospital of Stomatology, Cheeloo College of Medicine Shandong University & Shandong Key Laboratory of Oral Tissue Regeneration & Shandong Engineering Research Center of Dental Materials and Oral Tissue Regeneration & Shandong Provincial Clinical Research Center for Oral Diseases Jinan Shandong China

**Keywords:** angiogenesis, microtubule dynamics, nanosilicates, periodontal tissue regeneration, STAT3

## Abstract

Periodontal regeneration requires coupled angiogenesis and osteogenesis, while current strategies to promote angiogenesis face limitations such as poor cytokine stability and safety concerns. Nanosilicates (nSi), as bioactive nanomaterials with potent properties, show promise for enhancing bone regeneration via osteogenic pathways. However, their pro‐angiogenic potential and precise mechanisms, particularly within the periodontal microenvironment, remain poorly understood. This study addresses this knowledge void by introducing nSi into rat periodontal defects, revealing significantly enhanced vascular network formation and bone repair in vivo. Crucially, through intervention in relevant signalling pathways, this research provides the first evidence for the molecular mechanism underlying nSi‐induced angiogenesis in endothelial cells. We demonstrate that nSi regulate microtubule homeostasis via the MAPK‐mediated MAP4 signalling pathway, facilitating STAT3 nuclear translocation and ultimately promoting angiogenic differentiation. This mechanistic elucidation fills a critical gap in understanding the nSi–cytoskeleton–transcriptional regulation axis. These findings offer fundamental insights to guide the rational design and optimisation of nSi‐based biomaterial systems for vascularised periodontal regeneration.

## Graphical abstract

Nanosilicates triggered MAP4‐dependent microtubule remodelling via the ROS/MAPK pathway, shifting microtubules into a dynamic active state, which subsequently promoted activation of the STAT3 pathway, ultimately facilitating periodontal angiogenesis.

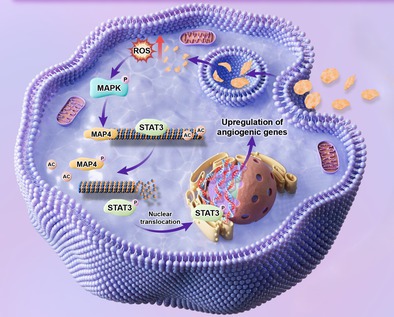

## 1 Introduction

Periodontitis, as a progressive inflammatory condition, is characterised by the destruction of periodontal supporting tissues, including gingival inflammation, attachment loss and subsequent alveolar bone resorption, ultimately resulting in tooth mobility and loss [1]. Admittedly, the desired outcome of periodontitis treatment is to achieve periodontal tissue regeneration, restoring both structure and function. Periodontal regeneration critically relies on the synergistic interplay of angiogenesis–osteogenesis coupling, wherein vascular networks function as dynamic channels of the elements for tissue regeneration, such as oxygen, signalling molecules and stem cells or immunomodulatory cells [2, 3]. The key determinant for establishing functional vascular networks is the angiogenic differentiation potential of endothelial cells, and modulating endothelial cell function is therefore vital for promoting periodontal angiogenesis and osteogenesis [4]. Conventional periodontal regeneration strategies, exemplified by guided bone regeneration, are often short of the capacity to release targeted bioactive signals crucial to actively orchestrate endothelial cell migration and angiogenic differentiation required for functional bone regeneration [5]. Currently, strategies like growth factor delivery or genetic regulation have been explored for promoting periodontal angiogenesis, which is hindered by several notable disadvantages, such as the poor protein stability of cytokines, unintended off‐target effects for genetic regulation, and the associated cost burden or safety concerns [6].

Recently, nanosilicates (nSi, Na^+^_0.7_[(Mg_5.5_Li_0.3_) Si_8_O_20_(OH)_4_]^−^_0.7_), emerging as innovative bioactive nanomaterials, have received widespread attention with potent biological effects and physicochemical properties. Compared to growth factors with short half‐lives and high‐dose requirements, nSi exhibit the characteristic of high stability under physiological conditions, and thereby take effect at low concentrations, translating into lower costs and potentially fewer side effects. Composed of 2D silicate nanoplatelets (diameter of 20–50 nm and thickness of 1–2 nm), nSi have exhibited significant potential in regenerative medicine stemming from their unique structural properties and bioactive ion release capacity (Na^+^, Mg^2+^, Si(OH)_4_, Li^+^) in aqueous solutions [2, 7, 8]. Recent studies have applied Mg^2+^ to bone healing based on its capacity to enhance endothelial cell function and promote angiogenesis. Existing research has confirmed the roles of nSi in enhancing bone regeneration, including the activation of osteogenic signalling pathways, whereas the pro‐angiogenic potential and the precise mechanisms of action in endothelial cells, particularly within the periodontal microenvironment, remain significantly understudied [9, 10, 11].

Signal transducer and activator of transcription 3 (STAT3) serves as a crucial transcription factor, regulating diverse physiological processes such as cell proliferation, apoptosis, and differentiation. STAT3 resides in the cytoplasm as an inactive form in its resting state. Upon stimulation by extracellular signals such as cytokines or hormones, STAT3 undergoes phosphorylation‐mediated activation, subsequently triggering its nuclear translocation, where it initiates the transcription of downstream target genes, including vascular endothelial growth factor (VEGF) [12, 13]. Crucially, the nuclear translocation of STAT3 is tightly orchestrated by the intricate dynamics of the microtubule cytoskeleton. Under basal conditions, when microtubules exist in a stable, polymerised configuration, STAT3 is sequestered in the cytoplasm through specific molecular interactions with microtubules. Upon specific signalling stimulation, microtubule depolymerisation releases the sequestered transcription factors, enabling their rapid diffusion into the nucleus [14]. The dynamics of microtubules are regulated by various proteins, such as microtubule‐stabilising proteins like microtubule‐associated protein 4 (MAP4). MAP4 can bind to tubulin, promoting microtubule polymerisation and thereby stabilising microtubules. However, phosphorylation of MAP4 causes it to dissociate from tubulin, shifting microtubules into a more dynamic state, which is associated with decreased levels of tubulin acetylation. Furthermore, activation of the p38 MAPK signalling pathway promotes the phosphorylation of MAP4, thereby altering microtubule dynamics [15, 16]. Consequently, it is hypothesised that nSi might achieve pro‐angiogenic regulation during the process of periodontal regeneration through the MAPK–microtubule–STAT3 axis, which remains an uncharted research territory.

In this study, nSi were loaded into the electrospinning fibrous membranes which were implanted into rat periodontal bone defect models. Our results demonstrated that nSi significantly enhanced vascular network formation and bone repair within the periodontal defect region. Crucially, we provided the first demonstration of the molecular mechanism underlying nSi‐promoted angiogenic differentiation in human umbilical vein endothelial cells (HUVECs): nSi regulate microtubule homeostasis through the MAPK‐mediated MAP4 signalling pathway, ultimately facilitating STAT3 nuclear translocation. The elucidation of the pro‐angiogenic mechanism of nSi would fill a critical research gap concerning the nSi–cytoskeleton–transcriptional regulation axis, thereby guiding the rational design and optimisation of nSi‐based biomaterial systems, especially for therapeutics promoting vascularisation during periodontal regeneration. Furthermore, this study holds significant implications for the development of novel vascularised periodontal tissue engineering scaffolds by enhancing intrinsic pro‐angiogenic activity, thereby overcoming the bottleneck of reliance on exogenous growth factors in current materials and boosting the cell‐free or minimally growth factor‐dependent periodontal regeneration strategies.

## 2 Materials and Methods

### 2.1 Materials Characterisations

Poly(lactic‐co‐glycolic acid) (PLGA), purchased from Jinan Daigang Biomaterial Co. Ltd., China, was used to produce the nSi‐loaded fibrous membranes by the electrospinning technique, and was dissolved in dichloromethane (DCM, Macklin, Shanghai, China) to prepare a 20% (w/v) matrix solution. A homogeneous 5% w/w dispersion of nSi in the matrix solution was achieved by sonication and stirring. Loaded into a metal‐needle‐fitted 5‐mL syringe, the spinning solution was electrospun towards an aluminium foil collector (18 cm needle‐to‐collector distance) at a flow rate of 2.5 mL/h and an applied voltage of 11–13 kV. Electrospinning proceeded at ambient temperature (50% relative humidity), followed by 14 days of vacuum desiccation of fibrous membranes to remove residual organic solvents. Employing the same fabrication procedure, we prepared two types of fibrous membranes, designated as PLGA and nSi‐PLGA.

The structure, elemental composition, and content of nSi were characterised using a JEM‐2100 transmission electron microscope (TEM; Jeol, Japan) coupled with energy‐dispersive spectroscopy (EDS) mapping. Surface morphology of the nSi‐loaded fibrous membranes was observed using an S‐4800 scanning electron microscope (SEM; Hitachi, Japan). Fibre diameters were quantified using Image J software (*n* = 100). Chemical composition analysis was performed by fourier transform infrared spectroscopy (FTIR; Thermo Scientific, USA).

### 2.2 Cellular Cytoskeleton Staining

The extraction and culture of periodontal ligament stem cells (PDLSCs), along with material sterilisation and pretreatment preceding cell inoculation, were performed according to our prior methodology [2]. The procedure of PDLSCs extraction has been approved by the Ethics Committee of Stomatological Hospital of Shandong University (Permit Number: 20220309). PDLSCs cultured for 3 days on the fibrous membrane underwent fixation (4% paraformaldehyde) and permeabilisation (0.5% Triton X‐100; Solarbio, Beijing, China). After blocking with 1% BSA (Sigma‐Aldrich), cells were treated overnight at 4°C with FITC‐phalloidin (Yeasen, Shanghai, China), followed by nuclear counterstaining using 2‐(4‐amidinophenyl)‐6‐indolecarbamidine dihydrochloride (DAPI; Solarbio). Cell morphology was assessed via confocal laser scanning microscope (CLSM; LSM 800, Carl Zeiss, Jena, Germany).

### 2.3 Animal Experiment

Periodontal defect models were established in male Wistar rats (8 weeks, SPF, Beijing, China), with approval from the Ethics Committee of Stomatological Hospital of Shandong University (Permit Number: 20211105). The rat anaesthesia was induced by intraperitoneal administration of pentobarbital sodium (40 mg/kg body weight). Subsequently, a critical bone defect (5 × 4 × 1 mm^3^; *L* × *H* × *D*) was surgically created in the mandibular buccal region, located 1 mm distal to the anterior mandibular margin and 1 mm apical to the alveolar ridge crest. Rats were randomly assigned to three groups receiving different membrane implants into the defects: (1) no treatment (Ctrl), (2) PLGA, or (3) nSi‐PLGA. Rats were euthanised at postoperative Week 2 through pentobarbital overdose followed by transcardial perfusion fixation using 4% paraformaldehyde. Mandibles were harvested for further experimentation.

### 2.4 Histological Analysis

Following decalcification of fixed samples in 10% disodium ethylenediamine tetraacetate (EDTA) at 4°C for 1 month, the specimens underwent dehydration in a graded ethanol series. After dimethylbenzene vitrification, specimens were paraffin‐embedded for the preparation of 5‐μm‐thick transverse sections from the defect area. Periodontal revascularisation and tissue regeneration were assessed by haematoxylin and eosin (H&E) staining and immunohistochemistry targeting CD31 and α‐smooth muscle actin (α‐SMA; Abcam, Cambridge, UK), performed in accordance with the manufacturer's protocols. Quantitative analysis of positive vascular rings and newly formed bone area was conducted using Image J software (NIH, Bethesda, MD, USA).

### 2.5 Cell Counting Kit‐8 Assay

Cytocompatible concentrations of nSi for the subsequent experiments were screened by cell counting kit‐8 (CCK‐8; Dojindo Laboratories, Kumamoto, Japan) assay following the manufacturer's instructions. HUVECs were incubated for 48 h in endothelial cell medium (ECM) containing graded nSi concentrations, followed by medium exchange for 10% CCK‐8/ECM solution. After 2.5 h of dark incubation at 37°C, optical absorbance at 450 nm wavelength was measured using a SPECTROstar Nano microplate reader (BMG Labtech, Offenburg, Germany).

### 2.6 TEM for HUVECs

Following 48 h of treatment with 100 μg/mL nSi, HUVECs were harvested via trypsinisation and centrifugation, then sequentially fixed with 2.5% glutaraldehyde and 1% osmium acid. After dehydration through an ascending ethanol series, the sample was permeated with an acetone–epoxy resin mixture, and then stepwise transitioned to pure epoxy resin. The samples were embedded in epoxy resin, polymerising under a graded temperature gradient. Subsequently, the cured resin blocks were sectioned into ultrathin slices of 60–80 nm, and underwent staining with 2% uranyl acetate aqueous solution for 20 min in the dark, and were observed by TEM.

### 2.7 Live/Dead Cell Staining

Live/dead cell staining for nSi‐treated HUVECs was performed using the Calcein AM/PI live/dead cell double stain kit (Solarbio). Following treatment with 25, 50 and 100 μg/mL nSi for 48 h, respectively, HUVECs were incubated with freshly prepared 2 μM Calcein AM staining solution for 20 min at 37°C, followed by 2 μg/mL PI staining solution for 5 min in the dark. After removing the staining solution, the cells were rinsed with phosphate‐buffered saline (PBS; Solarbio) three times and then observed under a fluorescence microscope (Leica DMi8, Wetzlar, Germany).

### 2.8 Tubule Formation Assay

HUVECs were treated with nSi‐supplemented ECM (25, 50 or 100 μg/mL) for 3 days. Following this treatment, the cell tubule formation assay was conducted according to the manufacturer's instructions. Matrigel (Corning, NY, USA) was thawed overnight at 4°C. Pre‐cooled 48‐well plates were then coated with 100 μL/well of Matrigel and incubated at 37°C for 30 min. Cells were harvested by trypsinisation and centrifugation, and then seeded into Matrigel‐coated 48‐well plates. At 6, 24 and 48 h of the cultivation process, cellular tube formation was evaluated microscopically with an Olympus microscope (OLYMPUS IX73, Tokyo, Japan). The crucial angiogenic parameters, including node number, segment count, mesh quantity and total tubule length, were quantified using Image J software.

### 2.9 Quantitative Reverse Transcription Polymerase Chain Reaction

Total RNA was extracted from HUVECs using TRIzol reagent (Takara, Japan) following the manufacturer's protocol. RNA concentration and purity were then assessed using a NanoDrop 2000 spectrophotometer (Thermo Fisher Scientific, Waltham, MA, USA). Subsequently, RNA was reverse‐transcribed into complementary DNA using the PrimeScript RT reagent kit (TaKaRa) following the manufacturer's protocol. Gene expression of VEGF, placental growth factor (PGF), kinase insert domain receptor (KDR, also known as VEGF receptor 2 [VEGFR2]), and platelet‐derived growth factor (PDGF) was then quantified by quantitative reverse transcription polymerase chain reaction (qRT‐PCR) with SYBR Premix Ex Taq II (TaKaRa) on a LightCycler 96 system (Roche, Basel, Switzerland). Primer sequences used to amplify the target genes and the housekeeping gene glyceraldehyde‐3‐phosphate dehydrogenase (GAPDH) are provided in Table S1.

### 2.10 Immunofluorescent Staining

Following 15‐min fixation with 4% paraformaldehyde and 15‐min permeabilisation using 0.5% Triton X‐100, HUVECs underwent blocking with 10% normal goat serum after three PBS washes. After overnight incubation at 4°C with primary antibodies against VEGFR2, STAT3, and α‐tubulin (1:500; Abcam), the cells were washed with PBS and incubated in the dark (37°C, 1 h) with Alexa Fluor 594‐conjugated goat anti‐rabbit IgG secondary antibody and Alexa Fluor 488‐conjugated goat anti‐mouse IgG secondary antibody (1:500; Proteintech, Wuhan, China). Nuclei were counterstained with DAPI (Solarbio). Fluorescence images were captured using a Leica DMi8 system (Leica, Wetzlar, Germany) under light‐controlled conditions.

### 2.11 Extraction of Free Tubulin and Polymeric Tubulin

Microtubule stabilisation buffer (MTSB), containing 100 mM PIPES, 2 mM EGTA, 0.1 mM EDTA, 0.5 mM MgCl_2_, and 20% (v/v) glycerol, was prepared, mixed well, and adjusted to pH 6.8. Following PBS washing, cells were washed with MTSB and subsequently permeabilised using 0.1% Triton X‐100 diluted in MTSB, supplemented with 1% protease inhibitor cocktail (Solarbio). The resulting supernatant containing the free tubulin fraction was collected. Subsequently, cells were lysed directly in the tissue culture plates with RIPA buffer containing 1% protease inhibitor cocktail. Lysates were harvested by scraping and centrifuged at 4°C, 12,000 rpm. The resulting supernatant, representing the polymerised tubulin fraction, was collected.

### 2.12 Total Protein Isolation and Western Blotting

HUVECs underwent lysis in RIPA buffer supplemented with 1% phenylmethanesulfonyl fluoride (Solarbio) and 1% phosphatase inhibitor (Boster, Wuhan, China), and whole‐cell lysates were harvested by scraping. Following protein concentration measurement with a BCA Protein Assay Kit (Solarbio), equal protein aliquots (20 μg/lane) were separated by 10% SDS‐PAGE gels, followed by electro‐transfer of the separated proteins with different molecular weights onto PVDF membranes (Millipore, Billerica, MA, USA). The membranes were blocked in 5% non‐fat milk for 1 h, and then incubated with primary antibodies (rabbit anti‐STAT3, rabbit anti‐phospho‐STAT3, rabbit anti‐p38 MAPK, rabbit anti‐phospho‐p38 MAPK, rabbit anti‐MAP4, rabbit anti‐phospho‐MAP4, mouse anti‐tubulin, rabbit anti‐acetylated tubulin, rabbit anti‐VDAC, rabbit anti‐GAPDH, dilution ratio: 1:1000; Abcam) overnight at 4°C, followed by incubation with horseradish peroxidase‐conjugated secondary antibodies (Proteintech, Chicago, IL, USA). Protein bands were detected using enhanced chemiluminescence reagents (Millipore) and imaged with an extra‐sensitive chemiluminescence‐compatible system (Amersham Imager 600; GE Healthcare Life Sciences, Pittsburgh, PA, USA). Quantitative analysis of protein expression levels was performed using Image J software.

### 2.13 DCFH‐DA Fluorescence Probe

The DCFH‐DA fluorescence probe was used for detecting the intracellular reactive oxygen species (ROS) levels. Following treatment with 25, 50 and 100 μg/mL nSi for 48 h, respectively, HUVECs were incubated in the DCFH‐DA solution diluted with serum‐free culture medium at 37°C for 20 min. After washing with serum‐free culture medium three times, the cells were observed using a Leica DMi8 system.

### 2.14 Statistical Analysis

Intergroup differences were assessed with a two‐way *t*‐test employing GraphPad Prism software (version 6; MacKiev Software). Results are reported as the mean ± standard deviation, and statistical significance was deemed at *p* < 0.05.

## 3 Results

### 3.1 nSi‐Promoted Periodontal Angiogenesis and Tissue Regeneration

**FIGURE 1:**
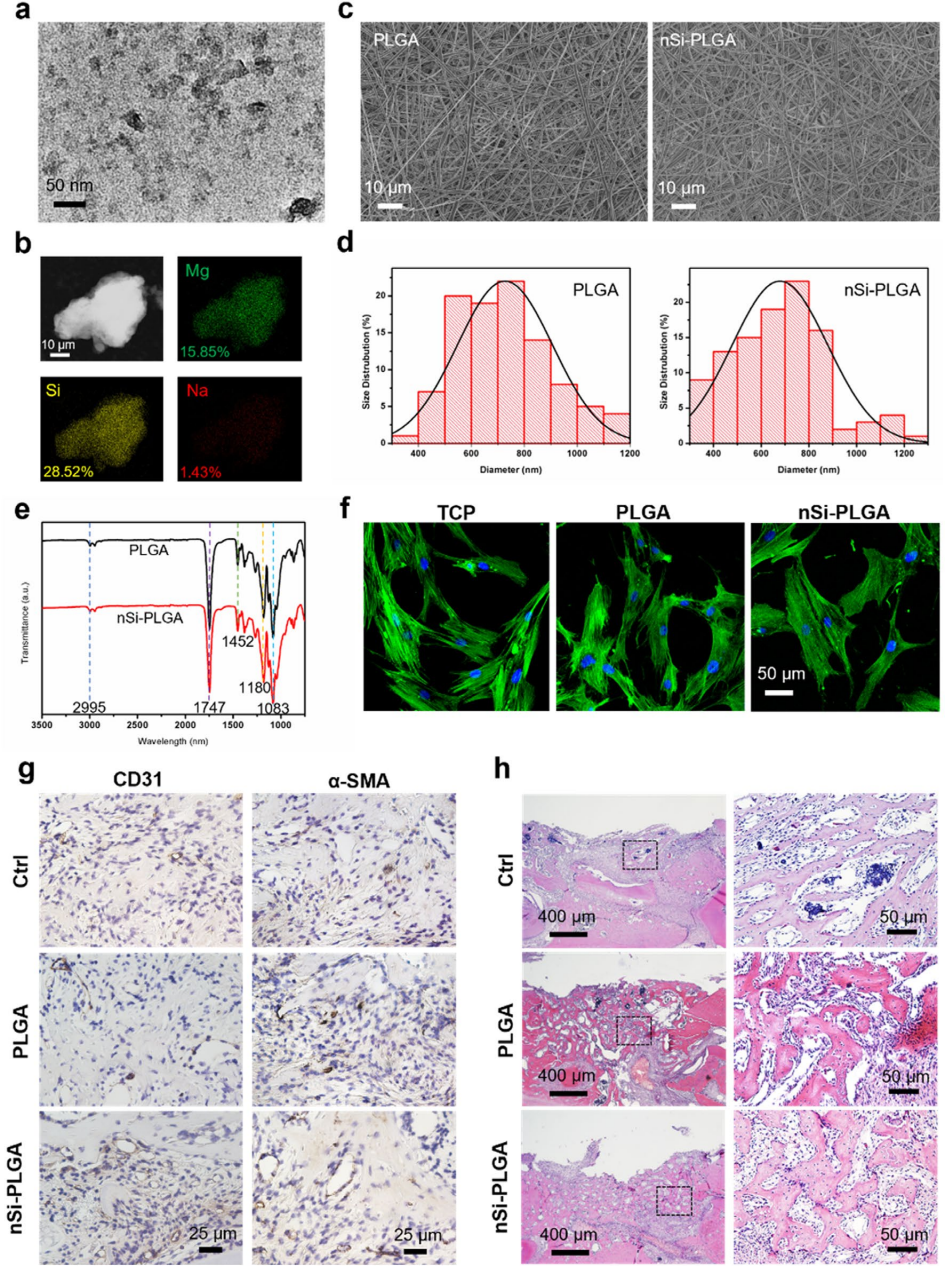
Characterisation and effect of nSi on angiogenesis in vivo. (a, b) TEM and EDS mapping images of nSi. (c, d) SEM images and diameter distribution analysis of nSi‐loaded membranes. (e) FTIR spectra of the membranes. (f) Cytoskeleton staining (green) by FITC‐phalloidin. (g) Immunohistochemical staining of CD31 and α‐SMA. (h) H&E staining of the periodontal defects. The visual fields framed by the black line were magnified.

nSi displayed a nano‐disk morphology with a diameter of approximately 50 nm in TEM imaging (Figure 1a). Elemental quantification via EDS mapping confirmed its elemental composition, with Na, Mg, Si and O weight percentages of 1.43%, 15.85%, 28.52% and 54.21%, respectively (Figure 1b). SEM characterisation revealed the surface morphology of the nSi‐loaded membranes with an interwoven fibrous structure (Figure 1c). The fibres exhibited smooth, uniform morphology without the blemishes of beads or fractures. nSi‐PLGA fibres showed reduced mean diameter (679 nm) compared to PLGA fibres (727 nm), indicating that nSi incorporation slightly reduced fibre diameter (Figure 1d). Surface chemistry and functional groups were analysed by FTIR spectroscopy (Figure 1e). The characteristic peaks identical to raw PLGA were identified as follows: peaks at 2995 and 2946 cm^−1^ correspond to –CH_3_ and –CH vibrations, respectively. The 1747 cm^−1^ absorption indicates carbonyl (–C═O) stretching. Ester –C–O stretching appears at 1180 and 1083 cm^−1^, while the 1452 cm^−1^ peak represents –CH_2_ bending. Peaks at 990 and 685 cm^−1^ are assigned to –CH bending vibrations. Cytoskeletal staining revealed the morphology of cells on the nSi‐loaded membranes (Figure 1f). Compared to cells on tissue culture plates, PDLSCs adhered to and fully spread along the material surface, exhibiting extended morphology, which suggested that this material did not adversely affect cellular adhesion or growth. The impact of nSi on periodontal revascularisation and tissue regeneration was verified with H&E staining and immunohistochemical analysis for angiogenesis‐related markers α‐SMA and CD31 (Figures 1g,h and S1). At 2 weeks post‐operation, nSi promoted angiogenesis in the periodontal defects, evidenced by increased round or oval brown‐stained structures, in comparison with other groups (Figure 1g, *p* < 0.05). Concurrently, nSi treatment generated significantly more abundant new bone formation relative to the other two groups (Figure 1h, *p* < 0.01). Collectively, these results confirm the role of nSi in enhancing periodontal angiogenesis and tissue regeneration in the early phases of wound healing in vivo.

### 3.2 nSi‐Enhanced Angiogenic Differentiation of HUVECs

**FIGURE 2:**
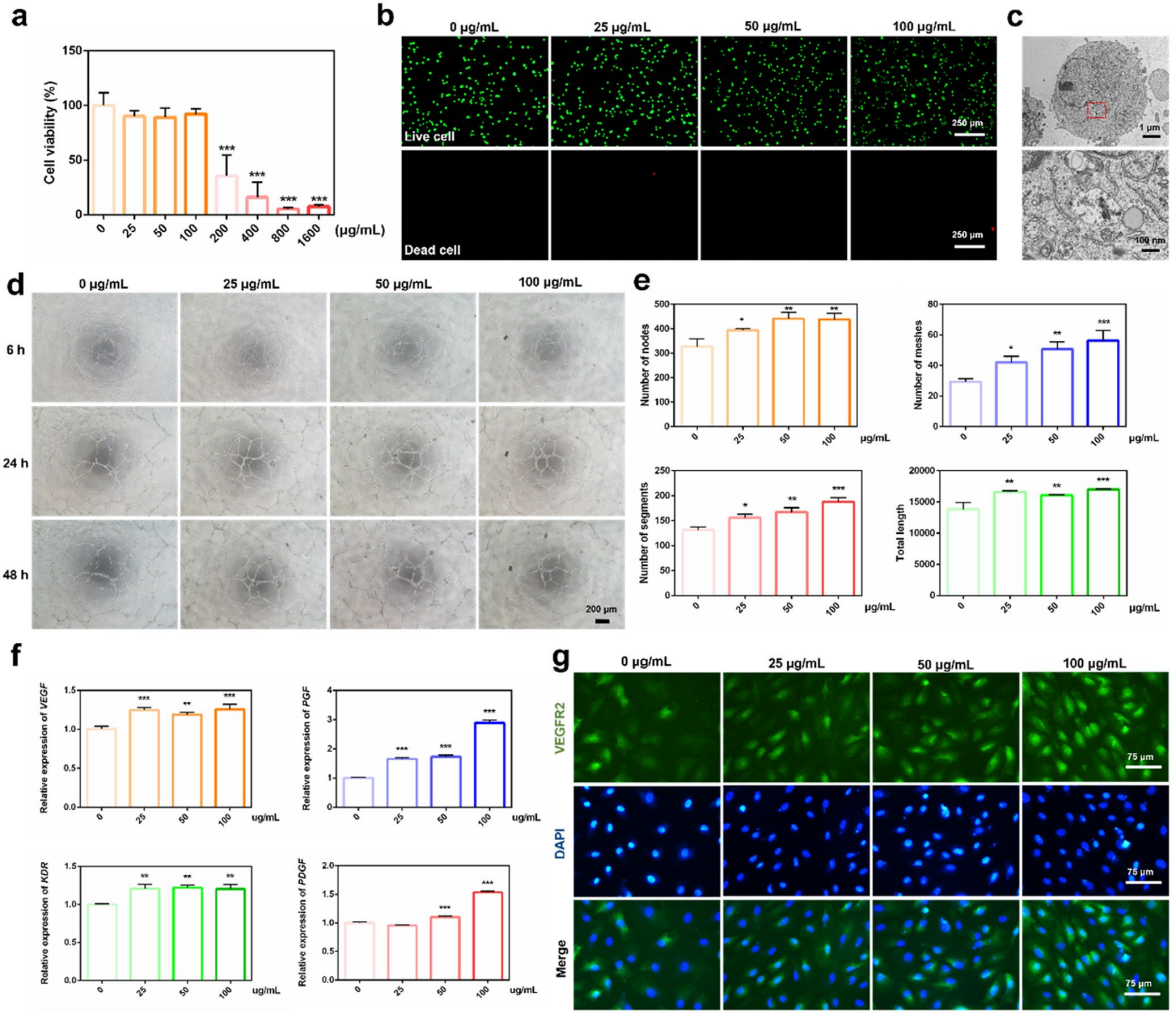
Angiogenic effect of nSi on HUVECs in vitro. (a) Cell viability after cultivation with different concentrations of nSi. (b) Live/dead cell staining. (c) TEM images for phagocytosis of nSi by HUVECs. (d) Representative images of the tubule formation assay of HUVECs cultured with nSi. (e) Quantitative analysis of the number of nodes, the number of segments, the number of meshes and the total tube length of the formed tubules. (f) Relative mRNA levels of angiogenesis‐related genes. (g) Immunofluorescent staining for VEGFR2.

CCK‐8 assay was performed to screen the cytocompatible concentrations of nSi for the subsequent experiments. The results indicated that obvious cytotoxicity occurred at concentrations above 200 μg/mL, with the relative cell viability of < 50% (Figure 2a). Furthermore, the live/dead staining showed that the proportion of living cells (green) after treatment with 25, 50 and 100 μg/mL nSi was similar to the control group (0 μg/mL) (Figure 2b). TEM image of nSi‐treated cells displayed that nSi could be phagocytosed by HUVECs (Figure 2c).

The angiogenic capacity of HUVECs treated with various concentrations of nSi was evaluated using a tube formation assay. While capillary‐like networks were formed in all HUVEC groups cultured on Matrigel for 6, 12 and 24 h, nSi treatment significantly enhanced tube formation in a concentration‐dependent manner (Figure 2d). Consistently, cells treated with 100 μg/mL nSi showed the highest values for nodes, segments, meshes and total tube length in quantitative analysis (Figure 2e, *p* < 0.05). Consistent with tube formation assay results, qRT‐PCR analysis revealed that various concentrations of nSi concentration dependently upregulated angiogenesis‐related gene expression (VEGF, PGF, KDR and PDGF; Figure 2f, *p* < 0.05). Furthermore, immunofluorescence staining detected VEGFR2 expression, indicating that 100 μg/mL nSi significantly enhanced VEGFR2 levels (Figure 2g). These results confirmed nSi's role in promoting angiogenesis in vitro. In addition, both the scratch assay and transwell migration assay showed that nSi significantly promoted cell migration in a concentration‐dependent manner (Figure S2).

### 3.3 nSi‐Induced Activation of Relevant Signalling Pathways in HUVECs

**FIGURE 3:**
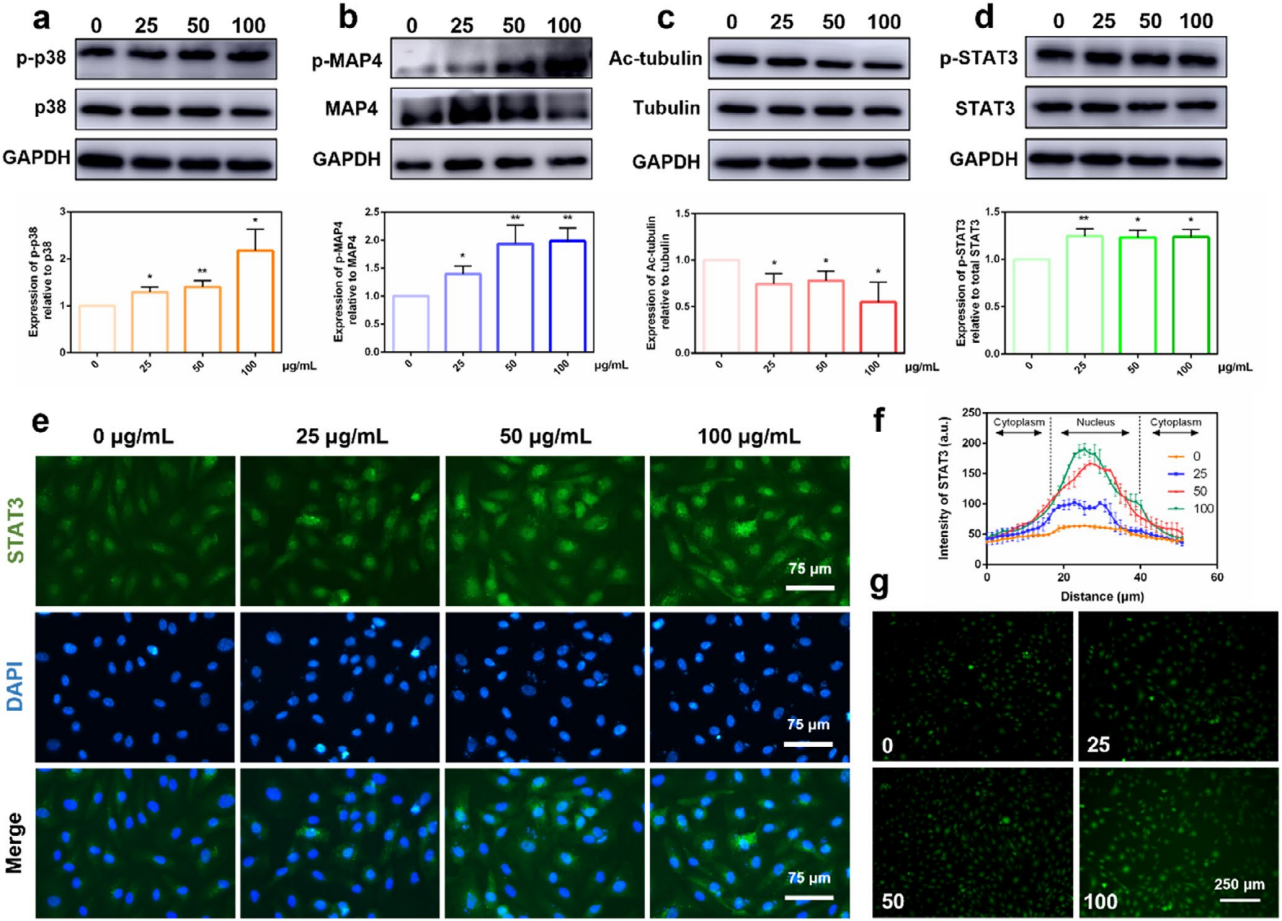
Activation of relevant signalling pathways in HUVECs cultured with nSi. (a) The phosphorylation level of the p38 MAPK pathway. (b) The phosphorylation level of MAP4. (c) The protein level of acetylated α‐tubulin. (d) The phosphorylation level of the STAT3 pathway. (e, f) The nuclear translocation of STAT3 (green fluorescent signals) and the corresponding quantitative analysis. (g) ROS level in HUVECs cultured with nSi.

Western blotting was performed to assess the activation of relevant signalling pathways by detecting the phosphorylation level of p38 MAPK, MAP4 and the STAT3 pathway, as well as the acetylation level of α‐tubulin. The results indicated that nSi facilitated the phosphorylation of p38 MAPK, MAP4 and STAT3, while reducing the acetylation of α‐tubulin in a concentration‐dependent manner (Figure 3a–d, *p* < 0.05). The activation of the STAT3 pathway was further evaluated by immunofluorescence staining, revealing that nSi concentration dependently promoted the nuclear translocation of STAT3 (Figure 3e,f). In addition, the intracellular ROS levels were detected using the DCFH‐DA fluorescence probe, consistently showing that nSi increased the intracellular ROS level, especially at a concentration of 100 μg/mL. Therefore, 100 μg/mL nSi was selected for subsequent mechanistic studies.

### 3.4 p38 MAPK Inhibitor Inhibited nSi‐Induced Angiogenic Effect and Activation of Downstream Signalling Pathways

**FIGURE 4:**
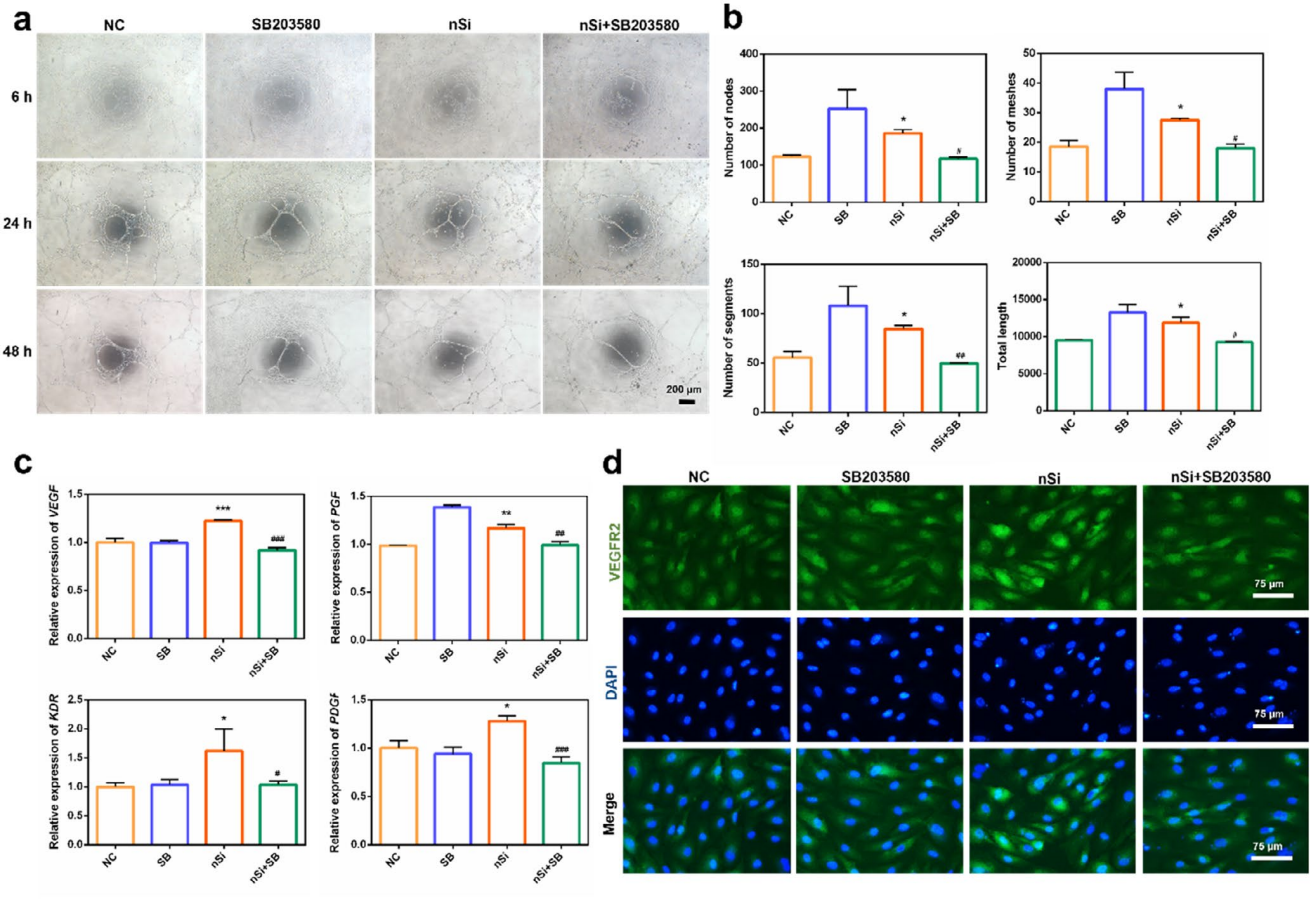
p38 MAPK inhibitor SB203580 inhibited the angiogenic effect of nSi. (a) Representative images of the tubule formation assay of HUVECs. (b) Quantitative analysis of the number of nodes, number of segments, number of meshes, and the total tube length of the formed tubules. (c) Relative mRNA levels of angiogenesis‐related genes. (d) Immunofluorescent staining for VEGFR2.

The potential involvement of the p38 MAPK pathway in regulating nSi‐mediated pro‐angiogenic effects was explored by using the inhibitor SB203580, the non‐cytotoxic concentration of which was screened by CCK‐8 assays (Figure S3a,b). SB203580 significantly inhibited capillary‐like network formation in nSi‐exposed HUVECs, with quantitative analysis demonstrating decreased nodes, segments, meshes, and total tube length (Figure 4a,b, *p* < 0.05). Furthermore, qRT‐PCR analysis indicated that SB203580 downregulated the expression of angiogenesis‐associated genes in nSi‐treated HUVECs (Figure 4c, *p* < 0.05). Likewise, VEGFR2 expression in HUVECs with nSi treatment was also attenuated by SB203580 (Figure 4d).

**FIGURE 5:**
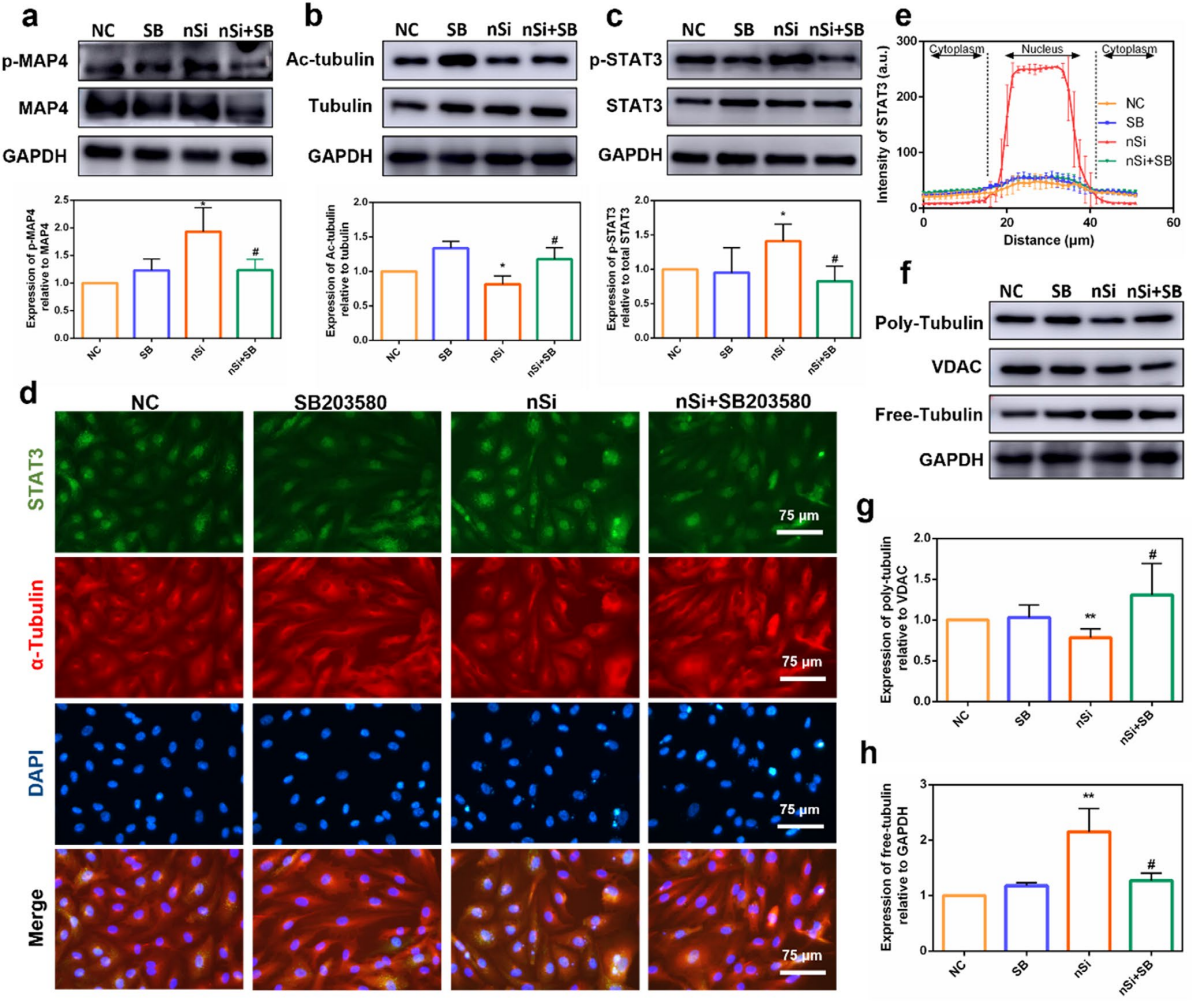
p38 MAPK inhibitor SB203580 inhibited the activation of downstream signalling pathways. (a) The phosphorylation level of MAP4. (b) The protein level of acetylated α‐tubulin. (c) The phosphorylation level of the STAT3 pathway. (d, e) The nuclear translocation of STAT3 (green fluorescent signals) and the corresponding quantitative analysis. (f–h) The protein level of polymerised and free tubulin.

Western blotting analysis demonstrated that nSi elevated MAP4 phosphorylation while suppressing α‐tubulin acetylation, and these modifications were antagonised by SB203580 treatment (Figure 5a,b). Furthermore, SB203580 attenuated nSi‐induced STAT3 pathway activation, evidenced by diminished STAT3 phosphorylation and nuclear translocation (Figure 5c,d). Supporting these results, immunofluorescence staining revealed that SB203580 diminished nuclear translocation of STAT3, concomitant with decreased tubulin depolymerisation (Figure 5d,e). Consistent with these observations, western blotting confirmed that SB203580 treatment partly reversed the nSi‐induced shift in microtubule equilibrium, namely the decline in polymeric tubulin and a concomitant rise in soluble tubulin (Figure 5f–h, *p* < 0.05).

### 3.5 Microtubule Dynamics Influenced nSi‐Induced Angiogenic Effect and Activation of STAT3 Pathway

**FIGURE 6:**
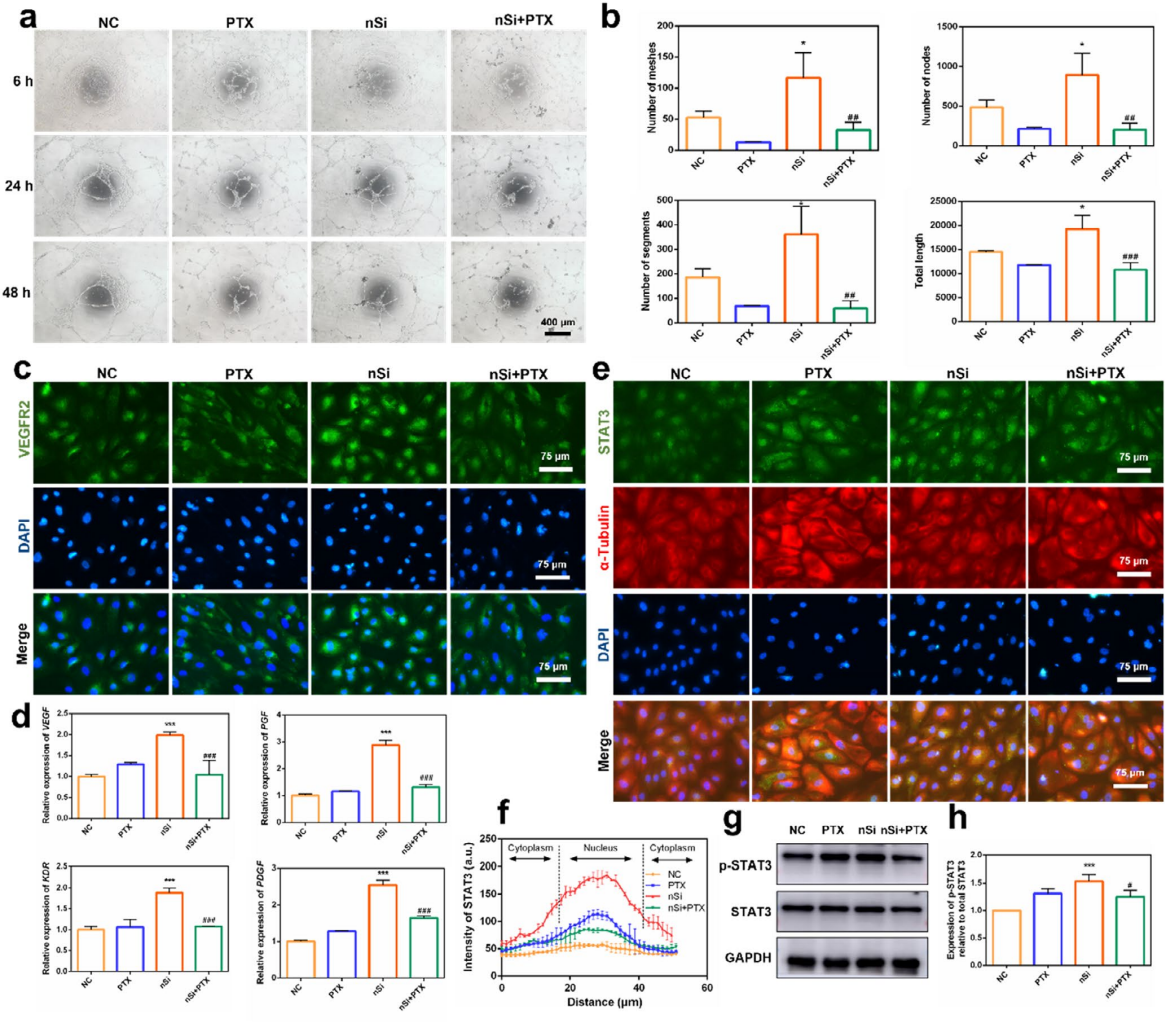
Microtubule stabiliser inhibited the angiogenic effect and the activation of downstream signalling pathways. (a) Representative images of the tubule formation assay of HUVECs. (b) Quantitative analysis of the number of nodes, the number of segments, the number of meshes and the total tube length of the formed tubules. (c) Immunofluorescent staining for VEGFR2. (d) Relative mRNA levels of angiogenesis‐related genes. (e, f) The nuclear translocation of STAT3 (green fluorescent signals) and the corresponding quantitative analysis. (g, h) The phosphorylation level of the STAT3 pathway and the corresponding quantitative analysis.

Microtubule equilibrium was disrupted using the microtubule‐stabilising drug paclitaxel, maintaining microtubule proteins in a polymerised state. Based on the CCK‐8 assay results, a non‐cytotoxic concentration of 10 μM paclitaxel was selected for subsequent experiments (Figure S3c,d). Paclitaxel significantly impaired capillary‐like network formation in nSi‐treated HUVECs, as quantified by reduced key metrics including nodes, segments, meshes, and total length (Figure 6a,b, *p* < 0.05). VEGFR2 expression in nSi‐treated HUVECs was also suppressed following paclitaxel treatment, consistent with the above findings (Figure 6c). qRT‐PCR analysis further demonstrated the downregulation of angiogenesis‐associated genes by paclitaxel in HUVECs with nSi treatment. (Figure 6d, *p* < 0.05). Paclitaxel suppressed nSi‐induced STAT3 pathway activation, manifested as reduced STAT3 phosphorylation and nuclear translocation (Figure 6e–h, *p* < 0.05). In agreement, immunofluorescence staining revealed that paclitaxel diminished nuclear translocation of STAT3, concurrent with an elevation in tubulin polymerisation (Figure 6e,f).

### 3.6 STAT3 Pathway Inhibitor Stattic Attenuated the Pro‐Angiogenic Effect of nSi

**FIGURE 7:**
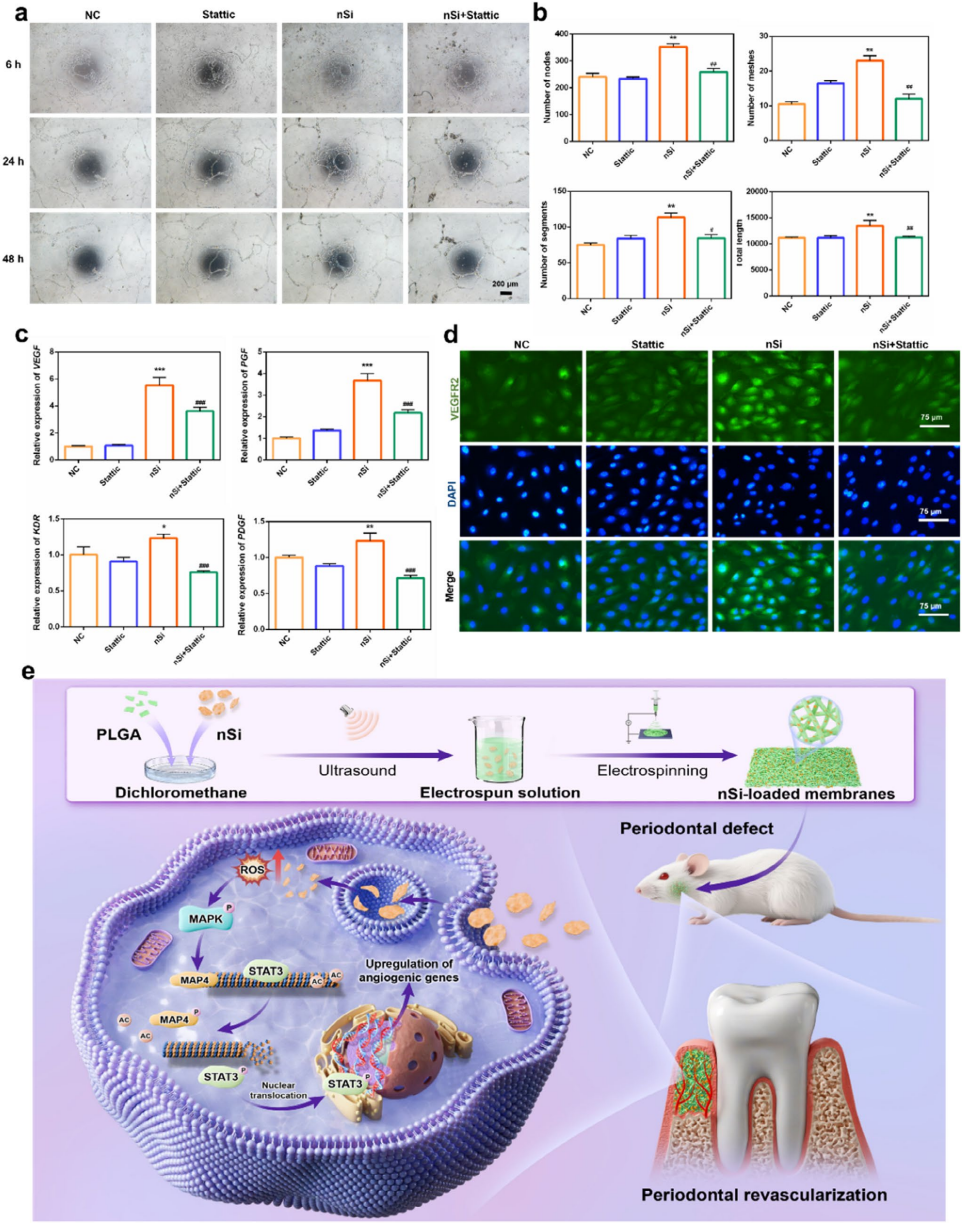
STAT3 inhibitor inhibited the angiogenic effect of nSi. (a) Representative images of the tubule formation assay of HUVECs. (b) Quantitative analysis of the number of nodes, number of segments, number of meshes, and the total tube length of the formed tubules. (c) Relative mRNA levels of angiogenesis‐related genes. (d) Immunofluorescent staining for VEGFR2. (e) Schematic diagram of the molecular mechanism of nSi.

The regulatory role of STAT3 signalling in nSi‐triggered pro‐angiogenesis was delineated via the specific pathway inhibitor stattic. The non‐cytotoxic concentration of stattic (10 μM) was determined by CCK‐8 assay for the subsequent experiments. (Figure S3e,f). Tube formation assay indicated significant suppression of capillary‐like networks by stattic in nSi‐treated HUVECs. Subsequent morphometric quantification confirmed obvious decreases in key parameters, including nodal points, vascular segments, meshed areas, and total tubule length (Figure 7a,b, *p* < 0.05). Consistent with the tube formation observations, transcriptional profiling by qRT‐PCR revealed the stattic‐generated suppression of angiogenic genes in nSi‐treated HUVECs (Figure 7c, *p* < 0.05). Reinforcing this regulatory effect, stattic significantly reduced VEGFR2 receptor expression in the same experimental model via immunofluorescent staining (Figure 7d).

## 4 Discussion

Our study explored the material characterisation and pro‐angiogenic effect of nSi for periodontal tissue regeneration using a periodontal bone defect model. Given that the underlying molecular mechanisms of nSi remained undetermined, we specifically focused on the regulatory roles of the MAPK–cytoskeleton–STAT3 transcriptional regulation axis in nSi‐induced pro‐angiogenic differentiation, and intervened in this signalling axis to screen for potential material action targets.

The TEM images presented the stacks of nSi, in which the sodium cation was shared between multiple nanoclay sheets owing to electrostatic interactions in dry environments [17, 18]. EDS analysis of TEM corresponded to the prior research [19], revealing a comparable nSi elemental profile consistent with the predicted formula Na^+^_0.7_[(Mg_5.5_Li_0.3_) Si_8_O_20_(OH)_4_]^−^_0.7_. While revealing smooth fibre morphology and homogeneous diameter distribution within the staggered framework, the nSi‐incorporated membranes showed slight diameter attenuation, an effect potentially mediated by nSi‐driven conductivity improvement [2]. FTIR spectra corroborated the retention of PLGA's functional groups and surface chemistry throughout electrospinning, as membrane peaks matched those of the raw material, which could shield cellular activities against interference by surface variability [20, 21]. Meanwhile, the biocompatibility of nSi‐loaded membranes was evidenced by cytoskeleton staining, revealing their capacity to serve as a biocompatible platform enabling cellular attachment and expansion.

Damaged alveolar bone regeneration is closely related to revascularisation of the defect area, wherein neovascularisation delivers vital components while concurrently permitting cell circulation critical for functional tissue repair [22, 23]. The osteogenic effect and relevant mechanisms of nSi have been well established [23, 24, 25], and our H&E staining results confirmed its efficacy in periodontal defect repairing, aligning with prior studies [26]. Crucially, we found that nSi enhanced angiogenic marker expression and new blood vessel formation within periodontal defects, as evidenced by immunohistochemical staining for α‐SMA and CD31. Nevertheless, the exact pathways mediating the pro‐angiogenic activity remain largely unexplored.

We observed that nSi can be internalised by HUVECs, which might be associated with clathrin‐mediated endocytosis, as reported in previous studies, followed by being transported to the lysosome for digestion and ultimately triggering related signalling cascades [18, 19]. In our study, nSi treatment significantly elevated the expression of angiogenesis‐related genes (VEGF, PGF, KDR and PDGF) in endothelial cells, concomitant with a marked enhancement in tube formation capacity and increased VEGFR2 expression. Furthermore, nSi promoted the phosphorylation of STAT3 at tyrosine residue 705 (Y705) and subsequent nuclear translocation, suggesting activation of the STAT3 signalling pathway. Under basal conditions, STAT3 adopts an inactive cytoplasmic localisation. Extracellular stimuli induce their phosphorylation‐dependent activation and subsequent nuclear translocation, initiating downstream target gene transcription. Empirical evidence has confirmed that this pathway could participate in angiogenesis by regulating VEGF expression [27, 28]. We found that the administration of the STAT3 inhibitor stattic significantly attenuated nSi‐induced pro‐angiogenic effects, indicating that STAT3 activation is a critical mechanism underlying nSi‐enhanced angiogenesis.

Microtubules, integral components of the cytoskeleton, are essential for cellular activities such as cell migration, vesicle transport and cellular development. Structurally, they are hollow cylinders consisting of 13 protofilaments, each formed by α‐tubulin/β‐tubulin heterodimers polymerised vertically in identical orientation [29, 30]. The nuclear translocation of transcription factors, such as STAT3, is dependent on microtubule dynamics. Under steady‐state conditions, STAT3 is sequestered in the cytoplasm by stabilised polymerised microtubules via specific molecular interactions. However, upon stimulation, microtubules undergo dynamic remodelling and release sequestered transcription factors during depolymerisation, allowing their rapid transport into the nucleus [14, 31, 32]. Microtubule acetylation is a cytoplasmic post‐translational modification of tubulin, occurring specifically at the Lys‐40 (K40) residue of α‐tubulin. Acetylated α‐tubulin is enriched in polymerised microtubules, thus establishing α‐tubulin acetylation as a marker of stable microtubule polymerisation [29, 30]. In this study, a significant decrease in acetylated α‐tubulin expression coupled with enhanced STAT3 nuclear translocation was observed upon nSi treatment, indicating that nSi could increase microtubule dynamic remodelling, driving microtubule dynamics into an active phase. Microtubule‐stabilising agent paclitaxel inhibits microtubule depolymerisation while elevating acetylated tubulin levels [33]. Following paclitaxel treatment, both the pro‐angiogenic effects of nSi and STAT3 pathway activation were attenuated. These findings suggest that nSi might facilitate STAT3 pathway activation and nuclear translocation through microtubule dynamics modulation.

Microtubule homeostasis is regulated by MAPs. MAPs promote microtubule polymerisation and enhance microtubule stability by binding to tubulin. As a MAPs family member, MAP4 is ubiquitously expressed across diverse cell types and tissues. The microtubule‐stabilising activity of MAP4 depends on its phosphorylation status: phosphorylation triggers MAP4 dissociation from microtubules, thereby allowing microtubules to enter a dynamic active state, concomitant with decreased levels of acetylated α‐tubulin. We observed nSi‐induced activation of the p38 MAPK pathway and phosphorylation of MAP4, which might align with the existing evidence that p38 MAPK activation triggers MAP4 phosphorylation [15, 16, 34]. Our findings also demonstrated that the administration of the p38 MAPK inhibitor SB203580 significantly suppressed nSi‐induced pro‐angiogenic differentiation in endothelial cells. Mechanistically, this suppression coincided with inhibition of MAP4 phosphorylation, increased microtubule polymerisation (evidenced by elevated acetylated α‐tubulin and polymerised tubulin, as well as reduced free tubulin), and blockade of STAT3 phosphorylation and nuclear translocation. These results confirmed the involvement of the p38 MAPK pathway in regulating nSi‐mediated pro‐angiogenic effects.

Moderately elevated intracellular ROS levels were also observed in nSi‐treated HUVECs using DCFH‐DA fluorescence assays. ROS, generated as by‐products of aerobic metabolism, trigger cytotoxicity, inducing apoptosis or cell death upon intracellular overaccumulation. At physiological concentrations, however, ROS function as crucial signalling mediators that orchestrate downstream MAPK pathway activity, thereby regulating cellular processes including proliferation, migration and differentiation [35, 36, 37, 38]. Consistent with these prior findings, nSi‐triggered activation of p38 MAPK signalling in HUVECs might be attributed to the moderately enhanced intracellular ROS levels. Consequently, our mechanistic investigation revealed that nSi triggered MAP4‐dependent microtubule remodelling via the ROS/MAPK pathway, shifting microtubules into a dynamic active state, which subsequently promoted STAT3 phosphorylation and nuclear translocation, ultimately facilitating angiogenic differentiation (Figure 7e).

## 5 Conclusion

Our study confirmed the pro‐angiogenic effect of nSi during the process of periodontal tissue regeneration. Crucially, we elucidated the underlying molecular mechanism: nSi activated the ROS/MAPK pathway to induce MAP4‐dependent microtubule remodelling, shifting microtubules into an active state. This cascade promoted STAT3 phosphorylation and nuclear translocation, thereby facilitating angiogenic differentiation. This mechanistic insight bridges the critical knowledge gap concerning nSi‐cytoskeletal signalling and transcriptional regulation, presenting valuable research concepts and potential intervention targets for advancing novel periodontal regeneration strategies, concurrently creating a substantial research basis for optimising nSi‐based material design and clinical applications.

## Author Contributions

Methodology, software, data curation, formal analysis, writing – original draft: Lingling Shang. Software, formal analysis: Yuhan Hu. Conceptualisation, writing – review and editing, funding acquisition, supervision: Shaohua Ge.

## Conflicts of Interest

The authors declare no conflicts of interest.

## Supporting information


**Figure S1:** Quantitative analysis of histological staining. (a) Percentage of newly formed bone. (b) Number of CD31^+^ and α‐SMA^+^ microvessel.
**Figure S2:** Pro‐migration effect of nSi on HUVECs. (a, b) Scratch test and the corresponding statistical analysis. (c, d) Transwell migration assay and the corresponding statistical analysis.
**Figure S3:** Cell viability after cultivation with different concentrations of drugs for 24 and 48 h. (a, b) p38 MAPK inhibitor SB203580. (c, d) Microtubule stabiliser paclitaxel. (e, f) STAT3 inhibitor stattic.
**Table S1:** Primer sequences for qRT‐PCR.

## Data Availability

The data that support the findings of this study are available from the corresponding author upon reasonable request.
