# Supplementary material for: Nanosilicates Enhanced Periodontal Angiogenesis by Regulating Microtubule Dynamic‐Mediated STAT3 Pathway
Source: Cell Prolif. 2025 Nov 4;59(5):e70143. doi: 10.1111/cpr.70143 (PMC13114780; doi:10.1111/cpr.70143)
Supplement: Supplementary file 1 — Figure S1: Quantitative analysis of histological staining. (a) Percentage of newly formed bone. (b) Number of CD31+ and α‐SMA+ microvessel. Figure S2: Pro‐migration effect of nSi on HUVECs. (a, b) Scratch test and the corresponding statistical analysis. (c, d) Transwell migration assay and the corresponding statistical analysis. Figure S3: Cell viability after cultivation with different concentrations of drugs for 24 and 48 h. (a, b) p38 MAPK inhibitor SB203580. (c, d) Microtubule stabiliser paclitaxel. (e, f) STAT3 inhibitor stattic. Table S1: Primer sequences for qRT‐PCR. [file CPR-59-e70143-s001.docx]

**Nanosilicates enhanced periodontal angiogenesis by regulating microtubule dynamic-mediated STAT3 pathway**

Lingling Shang, Yuhan Hu, Shaohua Ge*

Department of Periodontology, School and Hospital of Stomatology, Cheeloo College of Medicine, Shandong University & Shandong Key Laboratory of Oral Tissue Regeneration & Shandong Engineering Research Center of Dental Materials and Oral Tissue Regeneration & Shandong Provincial Clinical Research Center for Oral Diseases

Address: No.44-1 Wenhua Road West, 250012, Jinan, Shandong, China

***Corresponding Author:**

S. Ge, No.44-1 Wenhua Road West, 250012, Jinan, Shandong, China, Email: shaohuage@sdu.edu.cn

**
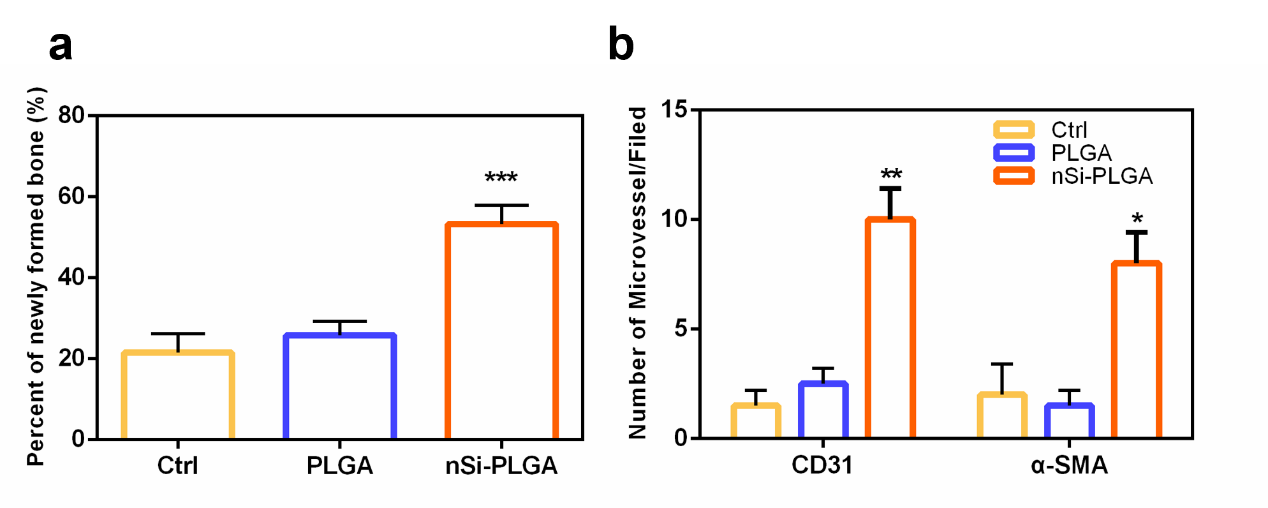
**

**Figure S1. Quantitative analysis of histological staining** (a) Percentage of newly formed bone. (b) Number of CD31+ and α-SMA+ microvessel.

**
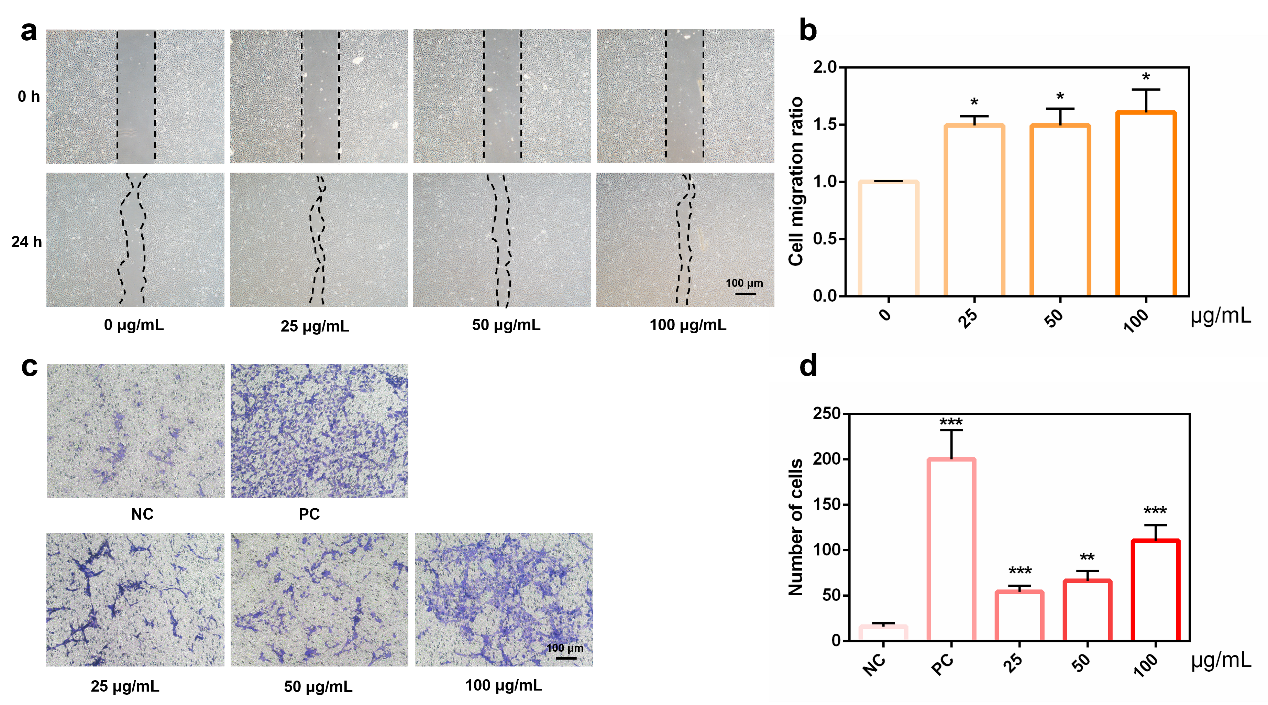
**

**Figure S2. Pro-migration effect of nSi on HUVECs** (a,b) Scratch test and the corresponding statistical analysis. (c,d) Transwell migration assay and the corresponding statistical analysis.


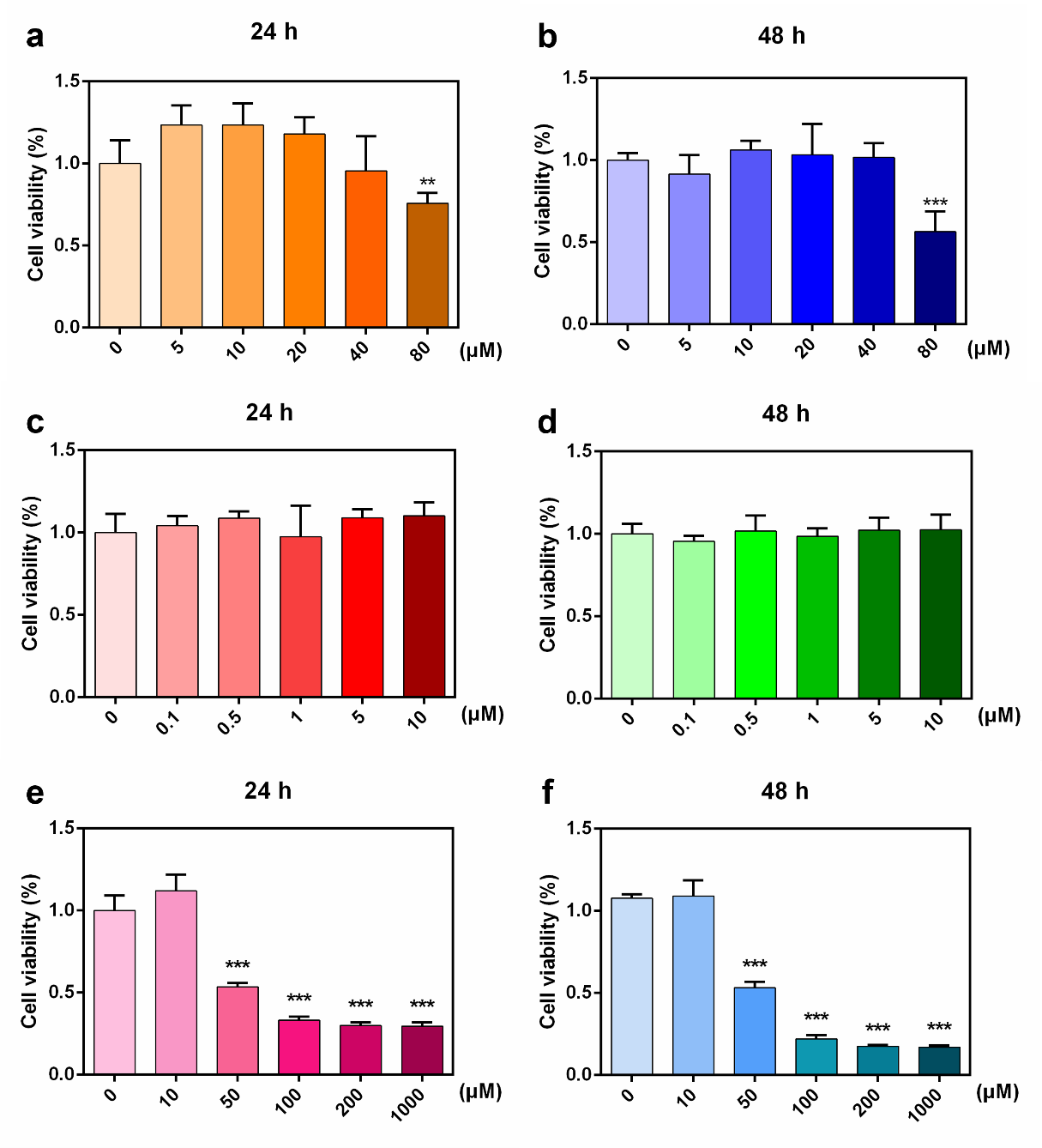


**Figure S3. Cell viability after cultivation with different concentrations of drugs for 24 h and 48 h.** (a,b) p38 MAPK inhibitor SB203580. (c,d) Microtubule stabilizer paclitaxel. (e,f) STAT3 inhibitor stattic.

**Table S1 Primer sequences for qRT-PCR**

| Gene | Forward (5′-3′) | Reverse (5′-3′) |
| --- | --- | --- |
| VEGF | **GGAGGGCAGAATCATCACGAA** | **GGTCTCGATTGGATGGCAGT** |
| KDR | **GGTTGTGTATGTCCCACCCC** | **GAGTGGTGCCGTACTGGTAG** |
| PDGF | **CGTCCGCCAACTTCCTGAT** | **TGACACTGCTCGTGTTGCAG** |
| PGF | **TTCAGCCCATCCTGTGTCTC** | **ATGGTCACATTGGCCGTCTC** |
| GAPDH | **GCACCGTCAAGGCTGAGAAC** | **TGGTGAAGACGCCAGTGGA** |
